# Effort–Reward Imbalance at Work and Drug Misuse: Evidence from a National Survey in the U.S.

**DOI:** 10.3390/ijerph182413334

**Published:** 2021-12-17

**Authors:** Jian Li, Timothy A. Matthews, Liwei Chen, Marissa Seamans, Constanze Leineweber, Johannes Siegrist

**Affiliations:** 1 Department of Environmental Health Sciences, Fielding School of Public Health, University of California, Los Angeles, Los Angeles, CA 90095, USA; tmatthews@ucla.edu; 2 School of Nursing, University of California, Los Angeles, Los Angeles, CA 90095, USA; 3 Department of Epidemiology, Fielding School of Public Health, University of California, Los Angeles, Los Angeles, CA 90095, USA; cliwei86@ucla.edu (L.C.); mseamans@ph.ucla.edu (M.S.); 4 Department of Psychology, Stockholm University, 114 19 Stockholm, Sweden; constanze.leineweber@su.se; 5 Institute of Medical Sociology, Faculty of Medicine, University of Düsseldorf, 40225 Düsseldorf, Germany; siegrist@uni-duesseldorf.de

**Keywords:** effort–reward imbalance, work stress, drug misuse, opioid, cannabis, workers

## Abstract

With the rise of drug misuse among workers in recent years, preliminary research on potential risk factors in the workplace of single-type of drug misuse has been reported. This is the first study to examine cross-sectional associations of work stress, in terms of effort–reward imbalance, with multiple drug misuse (including any drug misuse, opioid misuse, sedatives misuse, cannabis misuse, and other drug misuse) during the past 12 months in a national sample of U.S. workers. Data of 2211 workers were derived from the nationally representative and population-based Midlife in the United States (MIDUS) study. Internal consistency reliability and factorial validity of a 17-item effort–reward imbalance measure were robust and satisfactory. After adjustment for relevant covariates, logistic regression analyses showed that workers experiencing effort–reward imbalance at work had significantly higher odds of any drug misuse (OR and 95% CI = 1.18 (1.03, 1.37)), especially opioid misuse (OR and 95% CI = 1.35 (1.07, 1.69)) and other drug misuse (OR and 95% CI = 1.36 (1.01, 1.83)). The findings suggest that a stressful work environment may act as a determinant of drug misuse, and further prospective evidence is needed.

## 1. Introduction

Among the many risk factors of drug misuse and overdose, work-related conditions have received special attention in recent research. This holds particularly true for opioids [1,2,3,4,5], cannabis [6,7,8], and benzodiazepines [9,10]. Increased emphasis on this topic resulted from practical concerns, most obviously from the documented excess mortality of opioid overdose among working people [1,3,11]. Several of these studies explored distinct stressful aspects within the complexities of modern work environments and the results suggest that psychosocial hazards are more prevalent than physical hazards [12]. To this end, theoretical models are needed. These models are delineated at a level of generalization that allows for their identification in a wide range of different occupations and contexts. To this end, they focus on core aspects of working people’s crucial needs that are threatened by an adverse work environment, such as the need for security [13], the need for control and autonomy [14], or the need for reward and recognition [15]. Rooted in biopsychosocial stress theory [16,17], these models offer explanations of observed associations of adverse work environments with elevated health risks, including substance use and addiction. In this context, two models received special attention in international investigations: the demand–control model [14,18], and the effort–reward imbalance (ERI) model [15,19]. The former model, often labelled job strain, posits that job task profiles defined by a combination of high psychological demands and low control, or low degree of decision latitude, elicit sustained arousal with negative effects on a variety of health outcomes. The latter approach claims that lack of reciprocity between efforts spent at work and rewards received in turn is associated with strong negative emotions and sustained biological stress responses. In this model, rewards include salary or wage, job security, promotion prospects and esteem.

As it is difficult to clearly distinguish between medically prescribed drugs and drug misuse, several investigations testing these models focused on the former criterion. On balance, for both criteria, there is some evidence that these concepts of a stressful psychosocial work environment are related to an increased use of opioids [4,20], benzodiazepines [9], cannabis [21], and other psychotropic substances [10,22,23,24,25]. As a general limitation of current research on this topic, many studies examined just one type of drug misuse as the outcome. However, given the fact that drug misuse is often extended to a variety of substances [26,27], there is a need to investigate associations of stressful work with a comprehensive set of indicators of drug misuse. While two large-scale studies explored this relationship with indicators of the job strain model [28,29], a respective test based on the effort–reward imbalance, to our knowledge, has not yet been conducted. To fill this knowledge gap, we aimed to examine the hypothesis that effort–reward imbalance at work is associated with an elevated odds of reporting misuse of opioids, sedatives, cannabis, and other drugs, using data from a national survey among employed people in the United States (U.S.).

## 2. Materials and Methods

### 2.1. Study Population

Data from the second wave of the Midlife in the United States (MIDUS) [30] were used for this cross-sectional investigation. The MIDUS study is an ongoing nationally representative, population-based, longitudinal study examining psychological, social, and behavioral factors and health among U.S. adults aged 25–74 years. The second wave of the MIDUS survey was carried out from 2004 to 2006. Data collection was primarily based on random digit dial (RDD) phone interviews and an extensive self-administered questionnaire (SAQ). In total, 4963 people participated in the second wave of the MIDUS study, and 2313 reported that they were working. Among them, 2211 workers (95.6%) had complete data on ERI and drug misuse used for the current analyses.

### 2.2. Measures

The original ERI questionnaire [31] was not applied into the MIDUS study. Using existing items of psychosocial work characteristics, a proxy measure of ERI at work was constructed accordingly. It contains two 4-point Likert scales, “Effort” and “Reward”: ten items for “Effort”, including two subscales “Mental effort” (5 items) and “Physical effort” (5 items), and seven items for “Reward”, including three subscales “Job promotion” (4 items), “Esteem” (2 items) and “Job security” (1 item), corresponding to the structure of the original ERI questionnaire [31] (for details, please see the Table A1). For each scale or subscale, scores are determined by summing up point values for its items. Higher scores indicate higher effort and higher reward, respectively. Moreover, according to a predefined algorithm, a ratio between the two scales “Effort” and “Reward” was calculated to quantify the degree of mismatch between high ‘cost’ and low ‘gain’ at the individual level (weighed by numbers of items) [31].

The outcome of interest was drug misuse, which was defined according to the World Health Organization Composite International Diagnostic Interview short-form (CIDI-SF) [32], in accordance with the American Psychiatric Association Diagnostic and Statistical Manual of Mental Disorders, Revised Third Edition (DSM-III-R) [33]. The study participants were asked about their experiences during the past 12 months regarding ten types of drugs or substances (i.e., sedatives, tranquilizers, amphetamines, analgesics/prescription painkillers, anti-depressants, inhalants, marijuana/hashish, cocaine/crack/free base, hallucinogens, and heroin) “on your own”, which referred to “without a doctor’s prescription, in larger amounts than prescribed, or for a longer period than prescribed”. Under analgesics/prescription painkillers, a note stated “This does not include normal use of aspirin, Tylenol without codeine, etc., but does include use of Tylenol with codeine and other prescribed painkillers like Demerol, Darvon, and Percodan”. We followed prior established operationalizations of drug misuse in the MIDUS study [34]: analgesics/prescription painkillers and heroin were categorized as “opioid misuse”, sedatives and tranquilizers were combined into the category of “sedatives misuse”, marijuana/hashish as “cannabis misuse”, and the remaining drug types were grouped as “other drug misuse”; in cases where an individual reported any type of ten drugs, they were labelled as “any drug misuse”.

Several sociodemographic factors and health-related behaviors were included, including age (≤45; 46 to 55; and ≥56 years old), sex, marital status (married; never married; and others), race (white; black, and others), education (high school or less; some college; university or more), annual household gross income (<$60,000; $60,000 to $99,999; ≥$100,000), current smoking (no; and yes), alcohol consumption (low or moderate drinking—up to two drinks per day for men and one drink per day for women; heavy drinking—more than moderate drinking, [35]), and leisure-time physical activity (low; moderate; and high). Moreover, two potential mediators, pain and depressive affect, were also measured, given their significant impact on drug misuse [34]. Pain was considered as lower backaches and aches/stiffness in joints, depressive affect was determined by the CIDI-SF [34].

### 2.3. Statistical Analysis

Following descriptive analyses, we first used established procedures to test the psychometric properties of the proxy ERI measure. Cronbach’s alpha coefficients were calculated to assess internal consistency (values over 0.70 indicating satisfactory reliability). Factorial validity was tested with confirmatory factor analysis, which provided the closest representation of the theoretical structure, with two second-order factors “Effort” and “Reward” loading on a third-order factor representing the latent ERI construct, and with the components “Mental effort” and “Physical effort” identified as first-order factors loading on the “Effort” factor, as well as components “Job promotion”, “Esteem” and “Job security” identified as first-order factors loading on the “Reward” factor. We used the goodness-of-fit index (GFI), which indicates the amount of variance and covariance explained by the model (values over 0.90 indicating acceptable fit). Next, means and standard deviations (SDs) of ERI scales and subscales were computed, where we compared the differences between groups of drug misuse “No” and “Yes” using student’s *t* test. Finally, associations between all single scales and subscales of the ERI measure (increase or decrease per SD continuously) and drug misuse were estimated using logistic regression, and were expressed as odds ratios (ORs), with 95% confidence intervals (CIs). Multivariable models were calculated in three steps: Model I was adjusted for age, sex, marital status, race, education, and household income; further adjustment for smoking, alcohol drinking, and physical activity was added in Model II; Model III additionally adjusted for pain and depressive affect. We verified the fit of the logistic regression models with the Hosmer–Lemeshow goodness of fit test. In all cases, the models fit well (*p* > 0.05). All statistical analyses were performed with the program SAS 9.4 (SAS Inst., Inc., Cary, NC, USA).

## 3. Results

Table 1 shows the characteristics of the study sample. The 2211 participants in the sample were predominantly middle-aged, with a roughly equal proportion of men and women. Most participants were white and were married. The majority of participants had at least some college education. Most participants were non-smokers, had low or moderate alcohol drinking, and engaged in moderate-to high leisure-time physical activity. Approximately 30% and 7% of individuals reported pain and depressive affect, respectively.

**Figure 1:**
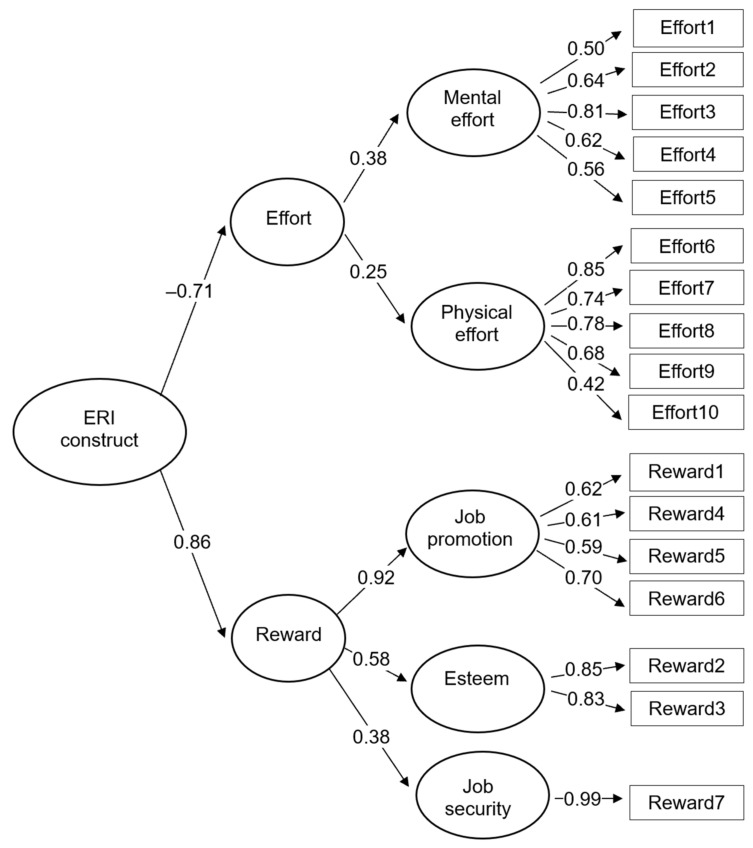
Confirmatory factor analysis testing the theoretical construct underlying the ERI measure.

The Cronbach’s alpha coefficients for the two scales of effort and reward were 0.74 and 0.76, respectively; for subscales of mental effort, physical effort, job promotion, and esteem, they were 0.76, 0.82, 0.72, and 0.83, respectively. Regarding factorial validity of the theoretical structure of the ERI measure, the third-order model represents a satisfactory fit to the data (e.g., GFI > 0.90) (see Figure 1).

The overall prevalence of any drug misuse was 11.5% in the MIDUS study subjects. Specifically, 3.9% with opioid misuse, 4.0% with sedatives misuse, 4.7% with cannabis misuse, and 2.4% with other drug misuse. The differences of ERI scales and subscales scores by drug misuse are given in Table 2. In general, individuals who reported drug misuse had significantly higher effort (especially physical effort), lower reward (especially job promotion), and a higher E-R ratio. Regarding the results of logistic regression analyses, as evident from Table 3, the series of adjusted models demonstrated several significant associations of effort–reward imbalance at work with drug misuse. In the fully adjusted model, taking the potentially mediating variables of pain and depressive affect into account, the theoretically important E-R ratio was associated with significantly elevated ORs of 1.18 (1.03; 1.37) for any drug misuse, 1.35 (1.07; 1.69) for opioid misuse, and 1.36 (1.01; 1.83) for other drug misuses. Among single scales, effort (in particular physical effort) was associated with any drug misuse and with cannabis misuse, while reward, specifically job promotion, was associated with opioid misuse and with other drug misuses. No associations were observed for sedatives misuse, and only weak relations with cannabis misuse.

## 4. Discussion

In this study, we observed that employed persons experiencing high effort and low reward at work had significantly higher odds ratios of opioid misuse, any drug misuse and other drug misuse (amphetamines, or anti-depressants, inhalants, or cocaine/crack/free base, or hallucinogens). No association was found for sedatives misuse, and cannabis misuse was uniquely related to the component of high physical effort. In subsequent models, effects were adjusted for a comprehensive set of confounding factors and remained robust. Given a cross-sectional study design, we cannot interpret the direction of associations between work stress and drug misuse. For instance, it is possible that drug use earlier on resulted in poor working and employment conditions, thus increasing the probability of experiencing stressful work in terms of this model [29]. In addition, we were not able to rule out that genetic or personality traits and maladaptive coping patterns predispose people to become susceptible to drug consumption, as well as the experience of sustained stress at work [36].

However, previous studies documenting prospective associations of effort–reward imbalance at work with long-term use of benzodiazepine [9] and of alcohol dependence [37] support the notion that stressful work may act as a determinant of drug misuse in this sample. Furthermore, this assumption is in line with the specific stress-theoretical link of this model to the pathways acting through limbic structures of the brain. These structures are sensitive to the experience and omission of reward [38]. As failed reward from social exchange violates basic expectations, thus threatening feelings of self-esteem and reinforcing a sense of being socially excluded, these structures may be activated in excessive ways [39]. Drug misuse offers a way of evoking positive emotional states of reward that buffer these threats [40]. The findings of this study corroborate earlier observations of associations of stressful psychosocial work environments with drug misuse, in particular those based on the job strain model mentioned above. Moreover, job insecurity and job loss were found to be related to excessive drug misuse, often resulting in premature mortality [1,3]. Nevertheless, prospective cohort studies are needed to validate this preliminary evidence.

It is of interest to note that effort–reward at work was not measured by the original questionnaire [31]. Yet, with a set of proxy indicators available from the MIDUS study, an appropriate assessment was achieved. Effort was assessed by 10 items, allowing the differentiation of mental and physical effort, and reward was measured by 7 items, covering the components of esteem, job promotion, and job security. Cronbach alpha values of scales and subscales, varying from 0.72 to 0.83, were acceptable, and confirmatory factor analysis replicated the theoretical structure of the model with good indices of fit. These psychometric properties of ERI measure in this U.S. sample are comparable to other validation studies [41], with extensively supportive evidence from European [31], Asian [42,43], and Latin-American countries [44]. Moreover, the ERI model has been used in a couple of studies in the U.S. to examine associations with different health outcomes; however, none of these studies covered general working people in this country, but instead investigated specific occupational strata, such as healthcare workers [45], firefighters [46], taxi drivers [47], cleaners [48], or older workers [49]. Taken together, the 17 items are considered a valid proxy measure of the two extrinsic scales of the effort–reward imbalance model in the U.S. working population, thus justifying the test of hypothesized associations. One critical remark relates to a higher number of items measuring physical effort, compared to the original measure. As two significant associations of this subcomponent with drug misuse were observed, one may ask whether this fact reduces the comparison with similar findings supporting the model’s hypotheses. It should be mentioned that a majority of significant associations in this analysis concerned the reward scale (five associations), in addition to the three theoretically prominent associations of the E-R ratio (see Table 3).

This study suffers from several limitations. In addition to its cross-sectional design, information on work stress, as measured by ERI, and drug misuse was collected by self-report, thus being potentially vulnerable to reporting bias. However, prospective cohort studies demonstrated successful predictions of health outcomes by ERI measures, thus supporting the validity of the measure [19]. Moreover, for large-scale epidemiological surveys, self-reported drug misuse under the framework of CIDI-SF and DSM-III-R is well established and validated [32,33]. The time window was restricted to the past 12 months, thus neglecting information on history of drug misuse over the previous life course [34]. The associations might be underestimated due to healthy worker survivor effect—the study participants must be healthy enough (not have debilitating addiction) in order to be employed. Furthermore, no data on personal vulnerability factors, social network constraints or devastating socio-environmental conditions stimulating drug misuse were included. Therefore, the available set of control variables included in logistic regression analyses cannot rule out the risk of confounding by unmeasured factors. Further adjustment for pain and depressive affect resulted in slight attenuation of associations between effort–reward imbalance and drug misuse, suggesting a potential mediating role of pain and depressive affect. Notably, much research evidence has accumulated in terms of effects of effort–reward imbalance at work on pain and depressive disorders [50,51], as well as effects of pain and depressive disorders on drug misuse [34,52,53]. We need to point out that the data source of our study came from the second wave of the MIDUS survey (2004–2006, with sample size 2211). Though data of the latest third wave (2013–2014) are available, the sample size of working people was reduced to approximately 1200. Considering the stability of the ERI structure in previous longitudinal studies [54], we preferred a larger sample to examine ERI psychometric properties. Finally, despite a fair representation of the original ERI measurement, this data set did not include the model’s intrinsic component ‘over-commitment’ and thus may slightly underestimate its explanatory contribution. Conversely, this study also exhibits some major strengths, as it adds to the small number of investigations that explore associations of stressful work with a comprehensive set of indicators of drug misuse, rather than focusing on a single outcome. Moreover, to our knowledge, this is the first study documenting consistent associations of a summary measure of the notion of effort–reward imbalance at work, the effort–reward ratio, with elevated probability of any drug misuse, of opioid misuse, and of other drug misuse. These elevated odds ratios remain statistically significant in the fully adjusted models. In addition, the MIDUS study offers a large, nationally representative, population-based sample comprised of workers in the U.S. across an extensive range of occupations, which is another distinct strength of this study.

## 5. Conclusions

In conclusion, preliminary evidence of associations between stressful work in terms of effort–reward imbalance and drug misuse, in particular opioid misuse, was observed, calling for further empirical support by prospective observational studies. Ideally, these studies apply the model’s original measurement tool and include objective data on drug misuse. In addition, specific vulnerability factors should be added to improve the accuracy of predictions. Once prospective findings corroborate the preliminary observation, worksite prevention approaches need to be developed to reduce future risks of drug misuse. Finally, given the scope of this problem, national policy measures of prevention and protection need to be adjusted to this new evidence.

## Figures and Tables

**Table 1:** Characteristics of the study sample (*N* = 2211).

Variables		*N* (%)
Age (years)	≤45	702 (31.75)
	46–55	827 (37.40)
	≥56	682 (30.85)
Gender	Men	1083 (48.98)
	Women	1128 (51.02)
Marital status	Married	1626 (73.55)
	Never married	194 (8.77)
	Others	391 (17.68)
Race	White	2036 (92.09)
	Black	72 (3.25)
	Others	103 (4.66)
Education:	High school or less	590 (26.68)
	Some college	629 (28.45)
	University or more	992 (44.87)
Annual household income (US $)	<60,000	820 (37.09)
	60,000–99,999	719 (32.52)
	≥100,000	672 (30.39)
Current smoking	No	1882 (85.12)
	Yes	329 (14.88)
Alcohol drinking	Low or moderate	2159 (97.65)
	Heavy	52 (2.35)
Leisure-time physical activity	Low	520 (23.52)
	Moderate	795 (35.96)
	High	896 (40.52)
Pain	No	1562 (70.65)
	Yes	649 (29.35)
Depressive affect	No	2047 (92.58)
	Yes	164 (7.42)

**Table 2:** Scores of effort–reward imbalance at work by drug misuse [means (SDs)].

	Drug Misuse: No	Drug Misuse: Yes	*p*
Any drug misuse	*N* = 1956 (88.47%)	*N* = 255 (11.53%)	
Effort	24.36 (5.19)	25.49 (5.04)	0.0011
Mental effort	12.90 (2.98)	12.90 (2.96)	0.9632
Physical effort	11.46 (3.97)	12.58 (4.08)	<0.0001
Reward	24.29 (3.42)	23.78 (3.58)	0.0269
Job promotion	13.65 (2.29)	13.32 (2.34)	0.0317
Esteem	7.11 (1.30)	6.94 (1.35)	0.0622
Job security	3.53 (0.83)	3.52 (0.81)	0.7633
E-R ratio	0.72 (0.22)	0.77 (0.24)	0.0002
Opioid misuse	*N* = 2125 (96.11%)	*N* = 86 (3.89%)	
Effort	24.42 (5.18)	26.08 (5.24)	0.0037
Mental effort	12.88 (2.98)	13.34 (3.00)	0.1633
Physical effort	11.54 (3.97)	12.74 (4.40)	0.0063
Reward	24.28 (3.42)	23.14 (3.88)	0.0027
Job promotion	13.64 (2.28)	12.84 (2.59)	0.0016
Esteem	7.10 (1.31)	6.82 (1.41)	0.0572
Job security	3.54 (0.83)	3.46 (0.84)	0.4425
E-R ratio	0.72 (0.22)	0.83 (0.32)	0.0017
Sedatives misuse	*N* = 2122 (95.97%)	*N* = 89 (4.03%)	
Effort	24.49 (5.20)	24.36 (4.82)	0.8096
Mental effort	12.92 (2.97)	12.42 (3.25)	0.1195
Physical effort	11.58 (4.010	11.94 (3.60)	0.3960
Reward	24.24 (3.45)	24.09 (3.40)	0.6884
Job promotion	13.61 (2.30)	13.55 (2.16)	0.7968
Esteem	7.09 (1.31)	6.96 (1.33)	0.3249
Job security	3.53 (0.83)	3.58 (0.76)	0.5459
E-R ratio	0.73 (0.22)	0.73 (0.21)	0.9916
Cannabis misuse	*N* = 2108 (95.34%)	*N* = 103 (4.66%)	
Effort	24.42 (5.20)	25.82 (4.65)	0.0079
Mental effort	12.91 (3.00)	12.67 (2.56)	0.3610
Physical effort	11.52 (3.98)	13.14 (4.06)	<0.0001
Reward	24.24 (3.45)	24.14 (3.27)	0.7913
Job promotion	13.62 (2.30)	13.44 (2.19)	0.4280
Esteem	7.08 (1.31)	7.16 (1.29)	0.5995
Job security	3.53 (0.83)	3.55 (0.76)	0.7915
E-R ratio	0.73 (0.23)	0.77 (0.19)	0.0895
Other drug misuse	*N* = 2159 (97.65%)	*N* = 52 (2.35%)	
Effort	24.45 (5.15)	26.06 (6.38)	0.0773
Mental effort	12.89 (2.98)	13.06 (3.16)	0.6956
Physical effort	11.56 (3.98)	13.00 (4.41)	0.0101
Reward	24.27 (3.43)	22.54 (3.64)	0.0003
Job promotion	13.64 (2.28)	12.54 (2.42)	0.0006
Esteem	7.10 (1.30)	6.54 (1.58)	0.0135
Job security	3.53 (0.83)	3.46 (0.78)	0.5327
E-R ratio	0.73 (0.22)	0.85 (0.31)	0.0078

Differences were determined by student’s *t* test; SD: standard deviation.

**Table 3:** Associations of effort–reward imbalance at work with drug misuse (odds ratios (ORs) and 95% confidence intervals (CIs)).

		Model I	Model II	Model III
Any drug misuse				
Effort	(increase per SD)	**1.20** (1.05, 1.38)	**1.19** (1.04, 1.37)	**1.16** (1.01, 1.34)
Mental effort	(increase per SD)	0.98 (0.85, 1.12)	0.97 (0.85, 1.11)	0.93 (0.81, 1.07)
Physical effort	(increase per SD)	**1.32** (1.14, 1.52)	**1.30** (1.13, 1.50)	**1.29** (1.12, 1.49)
Reward	(decrease per SD)	1.11 (0.98, 1.27)	1.11 (0.97, 1.27)	1.08 (0.95, 1.24)
Job promotion	(decrease per SD)	1.10 (0.97, 1.25)	1.10 (0.96, 1.26)	1.07 (0.94, 1.23)
Esteem	(decrease per SD)	1.11 (0.98, 1.26)	1.10 (0.97, 1.25)	1.08 (0.95, 1.23)
Job security	(decrease per SD)	1.01 (0.88, 1.15)	1.01 (0.88, 1.15)	1.00 (0.88, 1.14)
E-R ratio	(increase per SD)	**1.24** (1.08, 1.42)	**1.23** (1.06, 1.41)	**1.18** (1.03, 1.37)
Opioid misuse				
Effort	(increase per SD)	**1.30** (1.04, 1.63)	**1.30** (1.03, 1.62)	1.24 (0.99, 1.56)
Mental effort	(increase per SD)	1.17 (0.93, 1.46)	1.16 (0.92, 1.44)	1.10 (0.88, 1.38)
Physical effort	(increase per SD)	**1.28** (1.01, 1.62)	**1.28** (1.01, 1.61)	1.24 (0.99, 1.57)
Reward	(decrease per SD)	**1.29** (1.06, 1.57)	**1.29** (1.05, 1.57)	**1.23** (1.00, 1.51)
Job promotion	(decrease per SD)	**1.29** (1.06, 1.57)	**1.28** (1.05, 1.57)	1.23 (0.99, 1.50)
Esteem	(decrease per SD)	1.20 (0.98, 1.45)	1.20 (0.98, 1.46)	1.16 (0.96, 1.42)
Job security	(decrease per SD)	1.06 (0.86, 1.30)	1.06 (0.86, 1.30)	1.05 (0.86, 1.30)
E-R ratio	(increase per SD)	**1.43** (1.15, 1.78)	**1.43** (1.14, 1.78)	**1.35** (1.07, 1.69)
Sedatives misuse				
Effort	(increase per SD)	1.02 (0.82, 1.27)	1.02 (0.82, 1.28)	1.00 (0.79, 1.25)
Mental effort	(increase per SD)	0.89 (0.71, 1.10)	0.89 (0.71, 1.10)	0.84 (0.67, 1.06)
Physical effort	(increase per SD)	1.14 (0.90, 1.43)	1.14 (0.90, 1.43)	1.13 (0.90, 1.43)
Reward	(decrease per SD)	1.04 (0.83, 1.29)	1.03 (0.83, 1.28)	1.00 (0.80, 1.25)
Job promotion	(decrease per SD)	1.01 (0.81, 1.26)	1.01 (0.81, 1.26)	0.99 (0.79, 1.24)
Esteem	(decrease per SD)	1.13 (0.93, 1.38)	1.12 (0.91, 1.36)	1.09 (0.89, 1.34)
Job security	(decrease per SD)	0.90 (0.72, 1.13)	0.90 (0.71, 1.13)	0.89 (0.70, 1.12)
E-R ratio	(increase per SD)	1.05 (0.84, 1.32)	1.05 (0.84, 1.32)	1.01 (0.80, 1.27)
Cannabis misuse				
Effort	(increase per SD)	1.14 (0.92, 1.41)	1.09 (0.87, 1.36)	1.09 (0.87, 1.36)
Mental effort	(increase per SD)	0.83 (0.68, 1.03)	0.82 (0.66, 1.02)	0.81 (0.65, 1.01)
Physical effort	(increase per SD)	**1.38** (1.11, 1.72)	**1.32** (1.05, 1.66)	**1.32** (1.05, 1.66)
Reward	(decrease per SD)	0.94 (0.76, 1.16)	0.95 (0.77, 1.19)	0.95 (0.76, 1.19)
Job promotion	(decrease per SD)	0.98 (0.80, 1.21)	1.00 (0.81, 1.24)	1.00 (0.81, 1.24)
Esteem	(decrease per SD)	0.89 (0.71, 1.11)	0.89 (0.71, 1.11)	0.89 (0.71, 1.11)
Job security	(decrease per SD)	0.97 (0.78, 1.19)	0.99 (0.80, 1.22)	0.99 (0.80, 1.22)
E-R ratio	(increase per SD)	1.08 (0.87, 1.34)	1.05 (0.84, 1.32)	1.06 (0.84, 1.33)
Other drug misuse				
Effort	(increase per SD)	1.27 (0.96, 1.69)	1.24 (0.93, 1.65)	1.19 (0.89, 1.59)
Mental effort	(increase per SD)	1.05 (0.79, 1.40)	1.03 (0.78, 1.38)	0.95 (0.71, 1.28)
Physical effort	(increase per SD)	1.34 (0.99, 1.80)	1.30 (0.97, 1.76)	1.30 (0.97, 1.75)
Reward	(decrease per SD)	**1.42** (1.11, 1.82)	**1.42** (1.11, 1.82)	**1.38** (1.08, 1.78)
Job promotion	(decrease per SD)	**1.37** (1.07, 1.75)	**1.36** (1.06, 1.75)	**1.34** (1.04, 1.74)
Esteem	(decrease per SD)	**1.37** (1.09, 1.72)	**1.36** (1.09, 1.71)	**1.35** (1.07, 1.69)
Job security	(decrease per SD)	1.04 (0.80, 1.36)	1.05 (0.80, 1.37)	1.04 (0.79, 1.35)
E-R ratio	(increase per SD)	**1.44** (1.09, 1.92)	**1.43** (1.07, 1.91)	**1.36** (1.01, 1.83)

Logistic regression. Model I: Adjusted for age, gender, marital status, race, education, and income; Model II: Model I + additionally adjusted for smoking, alcohol drinking, and physical activity; Model III: Model II + additionally adjusted for pain and depressive affect.

## Data Availability

The raw data of the MIDUS study are publicly available from: https://www.icpsr.umich.edu/web/pages/NACDA/midus.html (accessed on 6 November 2021). The statistical SAS syntax supporting the conclusions of this article will be made available by the authors, without undue reservation. Requests to access the statistical SAS syntax should be directed to Jian Li, jianli2019@ucla.edu.

## Appendix A

**Table A1:** Proxy measures of effort–reward imbalance (ERI) at work.

Effort
(1) How often do you have to work very intensively—that is, you are very busy trying to get things done?
(2) How often do different people or groups at work demand things from you that you think are hard to combine?
(3) How often do you have too many demands made on you?
(4) How often do you have enough time to get everything done?
(5) How often do you have a lot of interruption?
(6) How often does your job require a lot of physical effort?
(7) How often does your job require you to lift loads weighing 50 pounds or greater?
(8) How often does your job require you to crouch, stoop, or kneel?
(9) How often does your job require you to stand for long periods of time?
(10) How often does your job require you to use stairs or inclines?
Mental effort: items 1, 2, 3, 4, 5; Physical effort: items 6, 7, 8, 9, 10 Response: All of the time/Most of the time/Some of the time/Rarely or Never
Reward
(1) To what extent do you feel cheated about the chances you have had to work at good jobs?
(2) To what extent do you feel a good deal of pride, when you think about the work you do on your job?
(3) To what extent do you feel that others respect the work you do on your job?
(4) To what extent do you feel that most people have more rewarding jobs than you do?
(5) To what extent do you feel that you’ve had opportunities that are as good as most people’s, when it comes to your work life?
(6) To what extent do you feel that it makes you discouraged that other people have much better jobs that you do.
(7) If you wanted to stay in your present job, what are the chances that you could keep it for the next two years?
Job promotion: items 1, 4, 5, 6; Esteem: items 2, 3; Job security: item 7 Response: A lot/Some/A little/Not at all
